# Extracellular vesicles released from microglia after palmitate exposure impact brain function

**DOI:** 10.1186/s12974-024-03168-7

**Published:** 2024-07-16

**Authors:** Gabriela C. De Paula, Blanca I. Aldana, Roberta Battistella, Rosalía Fernández-Calle, Andreas Bjure, Iben Lundgaard, Tomas Deierborg, João M. N. Duarte

**Affiliations:** 1 Department of Experimental Medical Science (EMV), Faculty of Medicine, Lund University, Sölvegatan 19, BMC C11, Lund, 221 84 Sweden; 2 Wallenberg Centre for Molecular Medicine, Lund University, Lund, Sweden; 3 Department of Drug Design and Pharmacology, Faculty of Health and Medical Sciences, University of Copenhagen, Copenhagen, Denmark

**Keywords:** Obesity, Neuroinflammation, Energy metabolism, Glycolysis, LPS

## Abstract

Dietary patterns that include an excess of foods rich in saturated fat are associated with brain dysfunction. Although microgliosis has been proposed to play a key role in the development of brain dysfunction in diet-induced obesity (DIO), neuroinflammation with cytokine over-expression is not always observed. Thus, mechanisms by which microglia contribute to brain impairment in DIO are uncertain. Using the BV2 cell model, we investigated the gliosis profile of microglia exposed to palmitate (200 µmol/L), a saturated fatty acid abundant in high-fat diet and in the brain of obese individuals. We observed that microglia respond to a 24-hour palmitate exposure with increased proliferation, and with a metabolic network rearrangement that favors energy production from glycolysis rather than oxidative metabolism, despite stimulated mitochondria biogenesis. In addition, while palmitate did not induce increased cytokine expression, it modified the protein cargo of released extracellular vesicles (EVs). When administered intra-cerebroventricularly to mice, EVs secreted from palmitate-exposed microglia in vitro led to memory impairment, depression-like behavior, and glucose intolerance, when compared to mice receiving EVs from vehicle-treated microglia. We conclude that microglia exposed to palmitate can mediate brain dysfunction through the cargo of shed EVs.

**Supplementary Information:**

The online version contains supplementary material available at 10.1186/s12974-024-03168-7.

## Introduction

Excessive consumption of diets rich in saturated fat leads to obesity that, together with its metabolic complications and comorbidities, can impact the brain [1–3]. In animal models, metabolic syndrome upon diet-induced obesity (DIO) has been reported to trigger hippocampal metabolic alterations, synaptic dysfunction, and impairments in learning and memory processes [4]. Interestingly, just as in animal studies, a relatively short exposure to a high-fat and high-sugar diet (four days) is sufficient to deteriorate hippocampal-dependent learning and memory in healthy humans [5].

Obesity is associated with a state of low-grade inflammation and, in the brain, DIO triggers neuroinflammation and microgliosis [6–11]. However, a controversy on neuroinflammatory processes induced by DIO can be found in the literature, such as a limited extension of gliosis and lack of cytokine overexpression in certain brain areas, such as hippocampus and cortex [12–16]. Moreover, cellular communication mechanisms other than cytokine release are likely to play a role in neuroinflammation in DIO, including the ability of microglia to shed eicosanoids and pro-resolving mediators [17], and extracellular vesicles (EVs) [18].

EVs are cell-derived membrane-surrounded vesicles (exosomes, ectosomes, microvesicles, apoptotic bodies, among others) that carry bioactive molecules (metabolites, nucleic acids and proteins) and, depending on their size, even cellular organelles. Thus, EVs participate in an orchestrated strategy of intercellular communication, including mediating inflammatory cues from microglia to other brain cells [19, 20] EVs are also transmitted across multiple bodily organs [21], although transfer across the blood-brain-barrier (BBB) might be restricted to particles of smaller size [22].

Palmitate, an abundant saturated fatty acid in diet, was found to increase in the cerebrospinal fluid of overweight and obese individuals relative to lean controls, and cerebrospinal palmitate concentration is correlated with body mass index and abdominal circumference, but not with obesity associated comorbidities, such as diabetes, dyslipidemia, or hypertension [23]. Melo et al. [23] further reported that intra-cerebroventricular (i.c.v.) injection of palmitate impairs synaptic plasticity and memory in mice, and increased astrocyte and microglia reactivity.

Using the BV2 microglia cell line, we investigated the gliosis profile and energy metabolism alterations induced by palmitate exposure, and tested the hypothesis that palmitate-exposed microglia utilize EVs as means of transmitting inflammatory messages to other cells.

## Materials and methods

### BV2 cells and treatment

Murine BV2 microglial cells (#CRL-2469, ATCC, Manassas, VA-USA; RRID: CVCL_0182) were cultured in T75 polystyrene flasks (#83.3911, Sarstedt, Nümbrecht, Germany) at 37 °C with an atmosphere of 5% CO_2_, using Dulbecco’s modified Eagle’s medium (DMEM), containing 5 mmol/L glucose, 1 mmol/L pyruvic acid, and 4 mmol/L glutamine (#11,885, Gibco, ThermoScientific, Göteborg, Sweden) supplemented with 10% fetal bovine serum (FBS, Gibco #10,500,064), 100 U/mL penicillin/streptomycin (Gibco #15,140,122). Cells were split every 2 days. For that, media was removed, cells were washed in Dulbecco’s phosphate-buffered saline without calcium and magnesium (DPBS, Gibco #14,190,250), and 1 mL of 0.05%(w/v) trypsin-EDTA with phenol red (Gibco #25,300,054) was added to detach the cells. After 5 min, 9 mL of fresh culture medium was added for re-seeding. For cell counting, suspended cells (10 µL) were mixed 1:1 with 0.4%(w/v) Trypan Blue (Sigma-Aldrich #T8154), the mixture was loaded in a hemocytometer, and live cells (unstained) were counted under a microscope.

For experiments, cells at passage 14–24 were seeded in polystyrene plates (unless otherwise stated) to a ~ 40% confluence as described below for each method. After 6 h, medium was replaced by DMEM without FBS for 12–16 h before treatment with either with vehicle, or palmitate (PA, 200 µmol/L) in 0.25%(w/v) bovine serum albumin (BSA) for 24 h (palmitate solution preparation as in supplementary methods). This palmitate concentration was selected from results of a dose-response pilot experiment, in which palmitate at 500 µmol/L for 24 h induced a cell detachment from the culture support above 50%. As a positive control of microglia reactivity, cells were incubated for 3 h with 1 µg/mL lipopolysaccharides (LPS) from *Escherichia coli* O111:B4 (Sigma-Aldrich #L2630; Lot #000110081) in 0.25%(w/v) BSA.

### Cell proliferation, viability and apoptosis

Cellular proliferation rates were determined at given time points by counting cells as described above. The CyQuant MTT Cell Viability kit (#V13154, Invitrogen, ThermoScientific) and the Caspase-Glo 3/7 assay kit (#G8090, Promega, Nacka, Sweden) were used in 96-well plates (Sarstedt #82.1581.001) to determine cell viability and apoptosis, respectively, following the manufacturer’s instructions. Experiments were performed in quadruplicates with seeding at a density of 10^4^ cells/well.

### Oxygen consumption and proton efflux rates

Cellular oxygen consumption rate (OCR) and proton efflux measured as extracellular acidification rate (ECAR) were analyzed in the Seahorse XF96 (Agilent, Santa Clara, CA-USA) following the manufacturer’s instructions (detailed in supplementary methods). Briefly, 10^4^ cells/well were seeded on the Seahorse cell culture plates using a total volume of 200 µL of culture medium. After 24 h, cells were incubated with the treatment solutions (treatments are described in the figure legends). OCR and ECAR values were normalized to total protein content, as determined with the bicinchoninic acid assay (kit from Pierce, #23,227, ThermoScientific).

### Quantitative real-time polymerase chain reaction (qPCR)

Cells were seeded onto 24-well plates (Sarstedt #83.3922.005) at a density of 5 × 10^4^ cells/well. After treatments, cells were washed with ice-cold phosphate-buffered saline (PBS, Gibco #18,912,014), treated with 100 µL trypsin as above, and re-suspended in 400 µL of culture medium. Total RNA was extracted with TRIzol (Invitrogen #15,596,026) following the manufacturer’s instructions, and RNA samples (500 ng) were reverse transcribed using the qScript cDNA Synthesis Kit (#95,047, QuantaBio, England). The resulting cDNA was used for qPCR with the reaction cocktail PerfeCTa SYBR Green FastMix (QuantaBio #95,072), and the primer pairs in Table 1. Reactions were run in duplicates, and relative gene expression levels were calculated with the ΔΔCT method using L14 as internal control gene.

**Table 1:** Nucleotide sequence of primers used for real-time PCR. These primers were previously validated by Greene et al. [69] and Skoug et al. [24]

Gene	Accession number	Forward Primer (5’→3’)	Reverse Primer (5’→3’)
*Il1b*	NM_008361.4	TGGACCTTCCAGGATGAGGACA	GTTCATCTCGGAGCCTGTAGTG
*IL6*	NM_031168.2	TACCACTTCACAAGTCGGAGGC	CTGCAAGTGCATCATCGTTGTTC
*Slc2a1*	NM_011400.4	CTTCATTGTGGGCATGTGCTTC	AGGTTCGGCCTTTGGTCTCAG
*Rpl14*	NM_025974.3	GGCTTTAGTGGATGGACCCT	ATTGATATCCGCCTTCTCCC
*Tnfa*	NM_013693.3	GGTGCCTATGTCTCAGCCTCTT	GCCATAGAACTGATGAGAGGGAG
*Ppargc1a*	NM_008904.3	TGATGTGAATGACTTGGATACAGACA	GCTCATTGTTGTACTGGTTGGATATG
*Opa1*	NM_001199177.2	TCTGAGGCCCTTCTCTTGTT	TCTGACACCTTCCTGTAATGCT
*Mff*	NM_029409.3	TCGGGTCTGTCCTCCCCATA	CAACACAGGTCTGCGGTTTTCA
*Mfn1*	NM_024200.5	TTGCCACAAGCTGTGTTCGG	TCTAGGGACCTGAAAGATGGGC
*Mfn2*	NM_133201.3	AGAGGCAGTTTGAGGAGTGC	ATGATGAGACGAACGGCCTC
*Dnm1l*	NM_001025947.3	TCACCCGGAGACCTCTCATT	TGCTTCAACTCCATTTTCTTCTCC
*Fis1*	NM_001163243.1	ACGAAGCTGCAAGGAATTTTGA	AACCAGGCACCAGGCATATT

### TNF-α ELISA

Media were centrifuged at 2,000 x g and 4 °C during 10 min to remove debris, and the supernatant was saved at -20ºC until use. Concentration of TNF-α was determined with a Mouse TNF-α ELISA kit (Abcam, #ab208348).

### Immunoblotting

Cells were seeded onto 6-well plates (Sarstedt #83.3920.005) at a density of 3 × 10^5^ cells/well, and treated as described above. Cells were washed with PBS, and proteins were extracted with a lysis buffer [in mmol/L: 150 NaCl, 1 ethylenediaminetetraacetic acid (EDTA), 50 tris(hydroxymethyl)aminomethane (Tris)-HCl, 1% (v/v) Triton X-100, 0.5% (w/v) sodium deoxycholate, 0.5% (w/v) sodium dodecylsulfate (SDS), pH 8.0] containing cOmplete protease inhibitors (Roche, Switzerland). After protein determination, we separated 30 µg of protein by SDS-PAGE and transferred onto nitrocellulose membranes as described elsewhere [24]. Membranes were blocked for 1 h at room temperature with 5%(w/v) BSA in Tris-buffered saline (in mmol/l: 20 Tris, 150 NaCl, and pH 7.6) containing 1% Tween-20 (TBS-T), incubated overnight at 4 °C with the OxPhos Complexes antibody cocktail (Invitrogen #45-8099), diluted 1:1250 in blocking solution. Membranes were then washed three times in TBS-T for 15 min and incubated for 2 h at room temperature with horseradish peroxidase-conjugated anti-mouse IgG secondary antibody (dilution 1:5000, Abcam #ab6789). After washing again, immunoblots were developed on the ChemiDoc (Bio-Rad, Sundbyberg, Sweden) using the SuperSignal West Pico PLUS Chemiluminescent substrate (ThermoScientific #34,580). For each independent experiment, the 3 experimental groups were run in parallel in the same gel, and signal from each band was normalized to the average of that in the 3 groups.

### Mitotracker staining and immunofluorescence microscopy

Cells (5 × 10^4^) were seeded onto a 35-mm glass-bottom dish coated with poly-D-lysine (#P35GC-1.5-14-C, MatTek, Ashland, MA-USA), and treated as described above. Then, cells were incubated for 30 min at 37ºC with 100 nmol/L of MitoTracker (Invitrogen #M7512; 1 mmol/L stock solution in DMSO). Stained cells were rinsed in PBS, and fixed with buffered 4% formaldehyde (Histolab, Askim, Sweden) for 15 min at room temperature. Fixed cells were rinsed 3 times, permeabilized during 10 min with 0.2%(v/v) Triton X-100 in PBS, blocked for 30 min in 5%(v/v) goat serum and 0.3%(v/v) Triton X-100 in PBS, and incubated overnight at 4°C with blocking solution containing the primary antibodies rabbit anti-allograft inflammatory factor 1 (Iba1, dilution 1:200; #019-19741, Fujifilm Wako, Japan) and mouse anti-β-actin (dilution 1:400, Sigma-Aldrich #A3854). Cells were then incubated for 1 h with the secondary antibodies AF488-conjugated anti-rabbit IgG (dilution 1:500, #AB150077, Abcam, Cambridge, UK) and AF647-conjugated anti-mouse IgG (dilution 1:500, Invitrogen #A21235), washed in PBS, mounted with 4,6-diamidino-2-phenylindole (DAPI)-containing Fluoroshield (Sigma-Aldrich #F6057), and imaged in a Nikon A1RHD confocal microscope with a CFI Apochromat TIRF 100 × Oil, NA 1.49 (Nikon Instruments, Tokyo, Japan). Images were acquired with NIS-elements (Laboratory Imaging, Nikon), and analyzed in ImageJ (NIH, Bethesda, MD-USA).

### ^13^C tracing experiments

Cells (2.2 × 10^6^) were seeded onto 10-cm dishes (#89089-612, ThermoScientific) and treated as above, using glucose-free DMEM (#11,966,025, ThermoScientific) supplemented with 5 mmol/L [1-^13^ C]glucose (99% C13 atom, Cortecnet, Voisins le Bretonmeux, France). [1-^13^ C]glucose was present during the 24 h of treatment with palmitate or vehicle, or for 21 h ahead and during the 3 h of LPS treatments. Cells were washed with ice-cold DPBS and frozen in N_2_ (l), and metabolites were extracted with 1 mL 80%(v/v) methanol. Then, samples were sonicated for 30 min at 4 °C, and centrifuged at 13,000 x *g*, 4℃, for 30 min. Supernatants were dried using a Savant SpeedVac (ThermoScientific) operating at room temperature and were saved for nuclear magnetic resonance spectroscopy (supplementary methods). Spectra were analyzed by line fitting using NUTS (Acorn NMR, Fremont, USA), as in previous studies [25]. Multiplet fractions from aliphatic carbons of glutamate were group-averaged and used in tcaCALC running on MATLAB 2019a (MathWorks, Natick, MA-USA) to determine rates (relative to citrate synthase) of pyruvate dehydrogenase (PDH), anaplerosis through pyruvate carboxylase (Y_PC_), anaplerosis through other pathways such as propionate or glutamine (Y_S_), and pyruvate kinase (PK) [26].

### EVs isolation

Cells were seeded at a density of 2 × 10^7^ in 75 cm2-flasks with 20 mL of medium. After treatment, EVs were obtained from culture medium as previously described [18]. Briefly, cell media were centrifuged at 400 x *g* for 5 min at room temperature to remove any cells. The obtained supernatant was centrifuged at 2,000 x *g* at 4 °C for 10 min to remove cell debris, and then at 10,000 x g at 4 °C for 30 min to remove large particles such as apoptotic cell bodies. The supernatant was again ultracentrifuged at 100,000 x *g* at 4 °C for 70 min to pellet the EVs. The resulting pellets were gently re-suspended in DPBS, and the ultracentrifugation step was repeated. Pelleted EVs were re-suspended in 20 µL of DPBS, and either kept at 4 °C overnight (for NTA and injection into the mouse brain) or stored at -20 °C (for proteomics).

### Nanoparticle tracking analysis (NTA)

EVs were analyzed using the NanoSight LM10 (Malvern Panalytical, Malvern, UK). Samples were diluted in PBS to 10^6^-10^9^ particles/mL and injected at 50 µL/min and at room temperature (21–22 °C). Particles were tracked 5 times during 60 s and analyzed with NanoSight NTA 3.4 (Malvern Panalytical) to determine particle diameter.

### Proteome analysis

Mass spectrometry (MS) was conducted as reported in supplementary methods. We adopted an all *versus* all contrast approach for the analysis of the protein signal intensities. Only proteins that were present in at least 2/3 independent experiments of each group were used for further analysis. A quantile-based regression was used to replace missing values with random draws. R version 3.6.2 (RRID: SCR_001905) was used for principal component analysis (PCA), and for differential enrichment testing using the package DEP: Differential Enrichment analysis of Proteomics data (https://rdrr.io/bioc/DEP). Significance thresholds were set to adjusted α = 0.05 and log_2_ of the fold change = 1. Significant findings were used for gene ontology analysis in using ShinyGO 0.77 (http://bioinformatics.sdstate.edu/go) and the Reactome pathway database.

### Animals

Sixteen 8-week-old male Swiss mice were obtained from Janvier Labs (Le Genest-Saint-Isle, France) and housed in groups of 4 under controlled conditions of humidity (55–60%) and temperature (21–23 ◦C) with a 12-hour light: dark cycle (lights on at 7:00). Chow and water were provided *ab libitum*. Mice were randomly selected (by coin tossing) to receive EVs either palmitate- or vehicle-treated BV2 cells in the lateral ventricle (supplementary methods), so that each cage housed 2 mice from either experimental group (total *n* = 8/group). Power calculations were not performed, but this sample size is sufficient to reveal DIO-induced memory impairment [11, 27]. Body weight was evaluated at the start and end of the study.

### Glucose tolerance test (GTT)

Food was removed for 5 h and, thereafter, blood glucose was measured from tail tip blood with the Accu-Chek Aviva glucometer (Roche, Manheim, Germany). Then, mice were given 2 g/kg glucose i.p. from a 30% (w/v) solution in saline, and blood glucose was measured from the tail tip at 15, 30, 60, 90 and 120 min after injection.

### Behavior

Mice were tested between 8:00 and 17:00, in a cubic open-field arena with side length of 50 cm, with room light adjusted to an illuminance of 15 lx. All experiments were recorded by an infrared camera into AnyMaze 6.0.1 (Stoelting, Dublin, Ireland). Mice were habituated to the experimental setup by exploring the empty arena during 5 min in 3 consecutive days. In the last habituation session, arena exploration was analyzed for total number of crossings between quadrants, time spent in center or perimeter (delineated at 8 cm from the walls), number of rearing events, and the clockwise and anti-clockwise rotations.

In the following day, memory performance was assessed with novel object recognition (NOR) and novel location recognition (NLR) tasks, as detailed previously [27] Briefly, mice were allowed to explore the arena for 5 min with two identical objects (familiarization phase). The mice returned to their home cage for 1 h (retention phase), and after were reintroduced for 5 min with one of the objects either replaced by a novel object or relocated in space (retention phase). The time spent exploring each object was analyzed in both familiarization and recognition phases.

The sucrose-plash test was used to evaluate depression-related behavior. Mice were sprayed with ~ 1 mL of a 10% sucrose solution on their dorsal coat and analyzed for latency between spraying and first grooming event, and total duration of grooming for 5 min [11].

### Immunofluorescence microscopy in brain slices

Animals under isoflurane anesthesia were transcardially perfused with ice-cold PBS, followed by 4% formaldehyde. Fixed brains were removed, embedded in 4% formaldehyde for 24 h, and cryoprotected in a 30% sucrose solution in PBS at 4 °C. Free-floating coronal Sect. (30 μm) were blocked for 2 h at room temperature with 5%(v/v) goat serum solution in PBS containing 0.3%(v/v) Triton X-100, and then incubated overnight at 4 °C with primary antibodies anti-Iba1 (1:200), and with anti-glial fibrillary acidic protein (GFAP) pre-tagged with AF488 (1:500; ThermoScientific #53-9892-82), or with anti-CD68 (1:500, Biorad #MCA1957). After washing with PBS, sections were incubated with the AF568-conjugated goat anti-rabbit IgG antibody (1:500, ThermoScientific #A21069) or AF647-conjugated donkey anti-rat IgG antibody (1:500, ThermoScientific #A78947) for 2 h at room temperature, washed again, and incubated with 4,6-diamidino-2-phenylindole (DAPI; 1 µg/mL in PBS; ThermoScientific #62,247) for 10 min. Slices were then mounted onto slides with ProLong Glass antifade medium (Invitrogen #P36980) and imaged in a A1RHD confocal microscope interfaced with NIS-elements (Nikon). Z-stack-projected images were processed and analyzed in ImageJ for area of Iba1, CD68 and GFAP immunoreactivity as previously described [16] Morphology of Iba1^+^ cells (3 cells/region/mouse) was analyzed with the AnalyzeSkeleton plugin to extract the number of cell processes and their length, and the number of branching points [28] One brain per group were excluded due to damage during processing.

### Statistical analysis

Researchers were blind to experimental group during analysis of behaviour videos and microscopy images. All data were analyzed using Prism 10.2.0 (GraphPad, San Diego, CA-US). Unless otherwise stated, data are presented as mean ± SD of n independent experiments. Normality was assessed with the Kolmogorov-Smirnov test, or the Shapiro-Wilk test for small sample sizes. Data not showing a Gaussian distribution were either analyzed with non-parametric tests, or log-transformed before ANOVA. Behaviour results deviating from a normal distribution are represented in boxplots extending from the 25th to 75th percentiles, line at median, and whiskers to the minimum and maximum values. Two-group comparisons were made with 2-tailed Student’s t-tests or Mann Whitney tests. Multiple groups were analyzed with either a Kruskal-Wallis test followed by Dunn’s multiple comparisons or with ANOVA followed by *post hoc* comparisons using the Holm-Šídák method. Significance was accepted for *P* < 0.05. All ANOVA results are available in Supplementary Table S1.

## Results

### Palmitate induces gliosis without cytokine overexpression

**Fig. 1:**
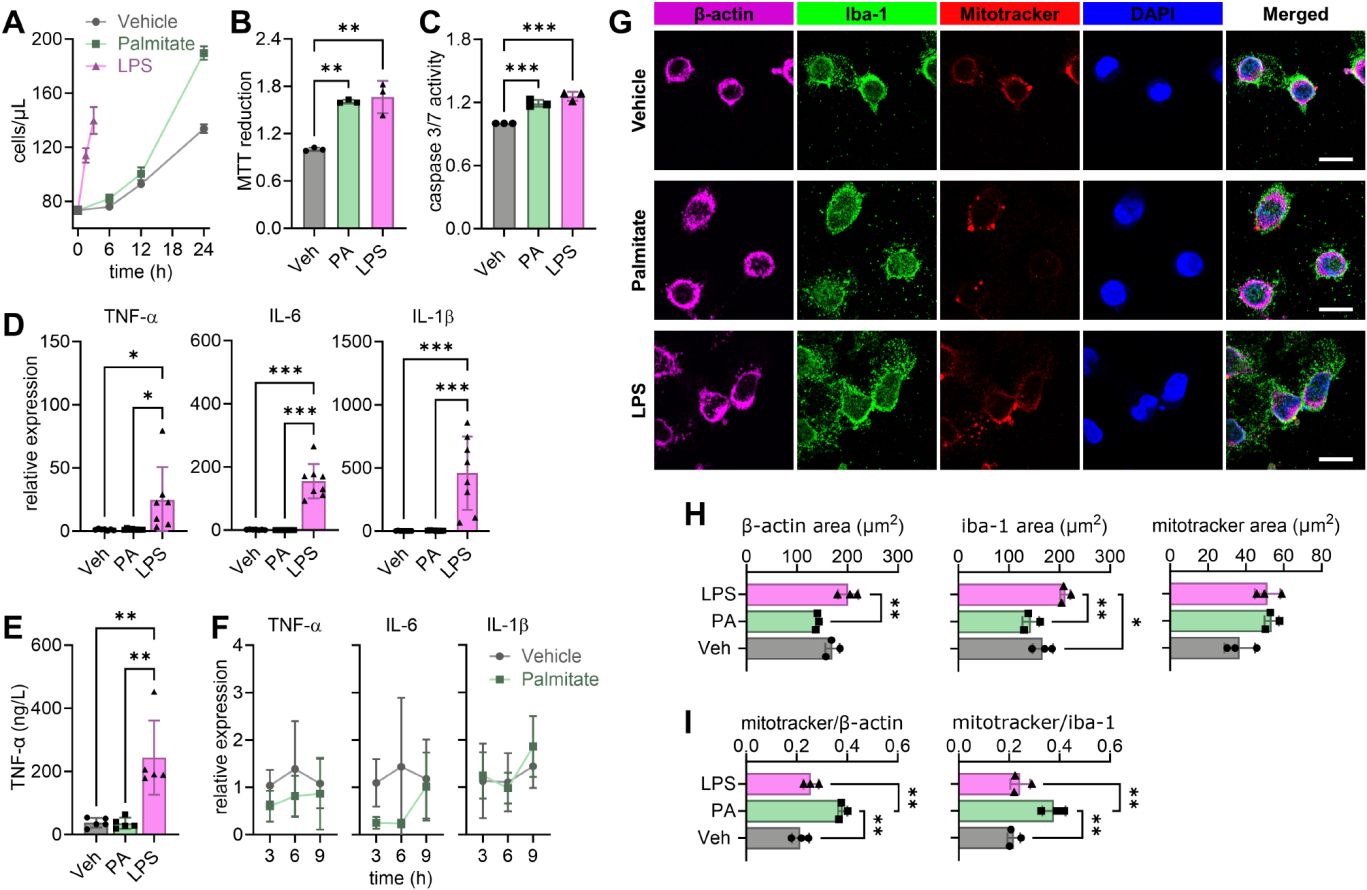
Palmitate exposure induces gliosis without exacerbated cytokine production and increases mitochondria content. BV2 cells were incubated with vehicle (Veh), 200 µmol/L palmitate (PA) for 24 h or 1 µg/mL LPS for 3 h. (**A**) Cell counts before, and after 6, 12 and 24 h of treatment, or after 1.5 and 3 h for LPS. (**B**) Relative cell viability measured by MTT reduction. (**C**) Relative activity of caspase 3/7. (**D**) Relative expression of TNF-α, IL-6 and IL-1β, and (**E**) concentration of TNF-α in the medium after treatment (3 h LPS, 14 h vehicle or palmitate). (**F**) Relative expression of cytokines during the initial 9 h of palmitate exposure. (**G**) Representative immunofluorescence micrographs for mitotracker, β-actin and Iba1 (scale bar is 10 μm). (**H**) Mean area occupied by mitotracker, β-actin and Iba1 signal per cell, and (**I**) area ratios of mitotracker to β-actin and to Iba1. Cells were analyzed within 3–4 fields of view from 3 independent experiments. Data is shown as mean ± SD of 3–8 independent experiments, represented by the individual symbols. *P < 0.05, **P < 0.01, ***P < 0.001 depict differences in comparisons following significant effects in ANOVA

BV2 cells responded to 200 µmol/L palmitate exposure during 24 h with increased proliferation, but at a slower rate than with LPS treatment (Fig. 1A), which was confirmed by an increased cell viability in the MTT assay (+ 66% for palmitate; +60% for LPS; Fig. 1B). There was also increased caspase activity, indicative of cell death or microglial activation [29], in palmitate- and LPS-treated cells relative to vehicle (+ 19% for palmitate; +26% for LPS; Fig. 1C).

Transcriptional levels of the cytokines TNF-α, IL-1β and IL-6, and levels of TNF-α released into the medium were unaffected by palmitate treatment, while significantly increasing upon LPS exposure, relative to vehicle-treated cells (Fig. 1D-E). We tested whether expression of cytokines was transiently modified at shorter palmitate exposure periods. Palmitate failed to increase cytokine expression at any of the time points assessed up to 9 h of exposure (Fig. 1F). Then, we set to determine whether palmitate expanded the area of the cells and their mitochondria, since palmitate can stimulate mitochondria metabolism and oxygen utilization in other cell models [30]. When compared to vehicle, palmitate-treated cells were of similar size, while LPS-treated cells increased the cell area as assessed by the immunoreactivity to either β-actin or the microglia marker Iba1 (Fig. 1G-H). The area of mitotracker staining was not substantially affected by either palmitate (*P* = 0.054) or LPS (*P* = 0.061) relative to vehicle (Fig. 1G-H). However, palmitate but not LPS exposure resulted in increased fraction of mitotracker fluorescence area relative to total cell area depicted by either β-acting or Iba1 (respectively, + 78% and + 73%; Fig. 1I).

Altogether, these findings suggest that BV2 microglia exposed to palmitate respond with increased proliferation and expanded mitochondrial density, but not with a typical neuroinflammatory response that involves cytokine overexpression.

### Distinct metabolism alterations after palmitate and LPS exposure

**Fig. 2:**
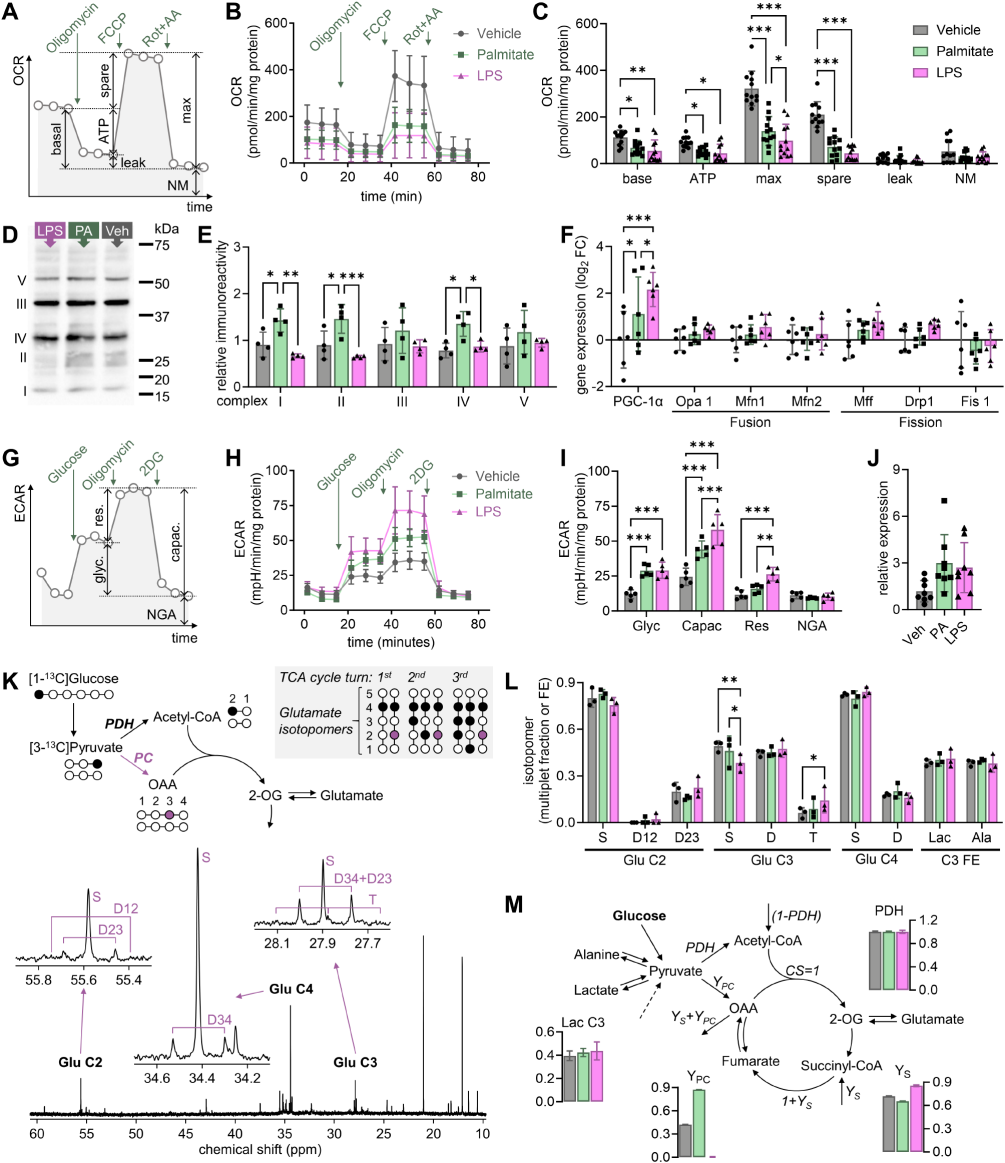
Palmitate exposure modulates energy metabolism in BV2 cells. BV2 cells were incubated with vehicle (Veh), 200 µmol/L palmitate (PA) for 24 h or 1 µg/mL LPS for 3 h. (**A**) Schematic representation of experiments for OCR measurements depicting the calculated parameters upon addition of oligomycin (1.5 mmol/L), FCCP (0.5 mmol/L), and antimycin A (0.5 mmol/L) plus rotenone (0.5 mmol/L): basal respiration (basal), proton leak-driven respiration (leak), ATP synthesis-linked respiration (ATP), maximal respiration capacity (max), spare respiration capacity (spare), and non-mitochondrial oxygen consumption (NM). (**B-C**) Oxygen consumption rate (OCR) measured for 3 cycles within each respiration state (**B**), and calculated respiration parameters (**C**). (**D**) Representative immunoblotting experiment against the four complexes of the electron transport chain and ATP synthase (complex V), after separation of 30 µg of protein by SDS-PAGE. (**E**) Relative immunoreactivity signal from the 5 complexes in 4 independent experiments. For a given protein, signal within each band was normalized to the average of that in the 3 experimental groups. (**F**) Expression of genes involved in mitochondria biogenesis, fusion and fission. (**G**) Schematic representation of experiments for ECAR measurements depicting the calculated parameters upon addition of oligomycin (1 mmol/L) and 2-deoxy-D-glucose (2DG, 50 mmol/L): basal glycolysis (glyc), glycolytic reserve (res), glycolytic capacity (capac), and non-glycolytic medium acidicitation (NGA). (**H-I**) extracellular medium acidification rate (ECAR) measured for 3 cycles within each respiration state, and calculated glycolytic parameters. (**J**) Relative expression of *Slc2a1* gene (GLUT1). (**K**) Representation of ^13^C incorporation into glutamate omitting, for simplicity, generation of isotopomers from unlabeled pyruvate/acetyl-CoA, and respective representative multiplets observed in ^13^C NMR spectra measured in extracts after metabolizing [1-^13^ C]glucose for 24 h. (**L**) Glutamate (Glu) multiplet fractions, and fractional enrichment (FE) of lactate C3 of (Lac) and alanine (Ala). (**M**) Model used in the TCAcalc analysis and relative fluxes, and lactate labeling estimated by fitting glutamate isotopomers, and Ala C3. Abbreviations: CS, citrate synthase; PDH, pyruvate dehydrogenase; Y, flux of anaplerotic substrates through pyruvate carboxylase (Y_PC_) or succinyl-CoA (Y_S_). Data is shown as mean ± SD of 3–12 independent experiments, represented by the individual symbols. *P < 0.05, **P < 0.01, ***P < 0.001 depict differences in comparisons following significant effects in ANOVA

Quiescent microglia mainly rely on oxidative phosphorylation for ATP production [31, 32]. Upon activation, microglia stimulate glucose uptake and glycolysis to meet enhanced energy demands [33, 34]. Thus, we set to measure palmitate-induced alterations of mitochondrial respiration. When compared to vehicle, cells exposed to either palmitate or LPS showed significantly lower baseline respiration, ATP-associated respiration, maximal respiration, and spare capacity (Fig. 2A-C). Proton leakage and non-mitochondrial oxygen consumption were similar across all groups (Fig. 2C). Since palmitate lowered mitochondrial respiration capacity, despite increased mitochondrial density, we then measured the density of mitochondrial respiration complexes and ATP synthase. Relative to vehicle-treated cells, palmitate but not LPS significantly increased the density of complexes I, II and IV by 57%, 63% and 73%, respectively (Fig. 2D-E). We further determined the expression of genes involved in mitochondrial dynamics and observed that both palmitate and LPS induced a significant increase in the expression of PGC-1α (Fig. 2F), which is a key regulator of mitochondria biogenesis. In turn, the expression of genes involved mitochondrial quality control by fusion (Opa1, Mfn1/2) and fission (Mff, Drp1, Fis1) was similar between the three groups (Fig. 2F).

Then, we analyzed ECAR as a surrogate of glycolysis (Fig. 2G-I). When compared to vehicle, both palmitate and LPS treatments increased basal glycolysis and the total glycolytic capacity, but only LPS increased the glycolytic reserve significantly (Fig. 2I). Non-glycolytic medium acidification was similar across treatments. We further measured the expression of *Slc2a1*, the gene encoding the glucose carrier GLUT1, which tended to be increased by palmitate (adjusted *P* = 0.065) and LPS (adjusted *P* = 0.097) treatments compared to vehicle (ANOVA *F*(2,21) = 3.54, *P* = 0.048; Fig. 2J).

Finally, we performed ^13^C metabolic tracing to infer on the rearrangement of fluxes through anaplerotic pathways. After treatment, cells were allowed to metabolize [1-^13^ C]glucose for 24 h, and metabolite extracts were analyzed by ^13^C NMR spectroscopy to determine labeling of glutamate carbons (Fig. 2K). Glutamate (Glu) multiplet fractions, were mainly affected by LPS when compared to vehicle, namely Glu C3 (Fig. 2L). However, there were negligible modifications in the labeling of individual multiplets for palmitate *versus* vehicle. A metabolic flux analysis using TCAcalc estimated that, relative to the citrate synthase flux, pyruvate carboxylation was higher in palmitate-treated than vehicle and blunted in LPS treated cells (Fig. 2M). LPS-treated cells had, instead, faster rate of anaplerosis feeding succinyl-CoA than the remaining groups. Lactate labeling was a fitted parameter in the model, and the result correlated well with that measured experimentally (Pearson *r* = 0.9998, *P* = 0.014).

In sum, the increased proliferation induced by palmitate is accompanied by a rearrangement of energy metabolism fluxes, namely increased glycolysis, reduced mitochondrial respiration, and increased pyruvate carboxylation that can support *de novo* oxaloacetate synthesis for replenishment Krebs cycle intermediates used in biosynthetic pathways.

### Microglia communication via EVs

**Fig. 3:**
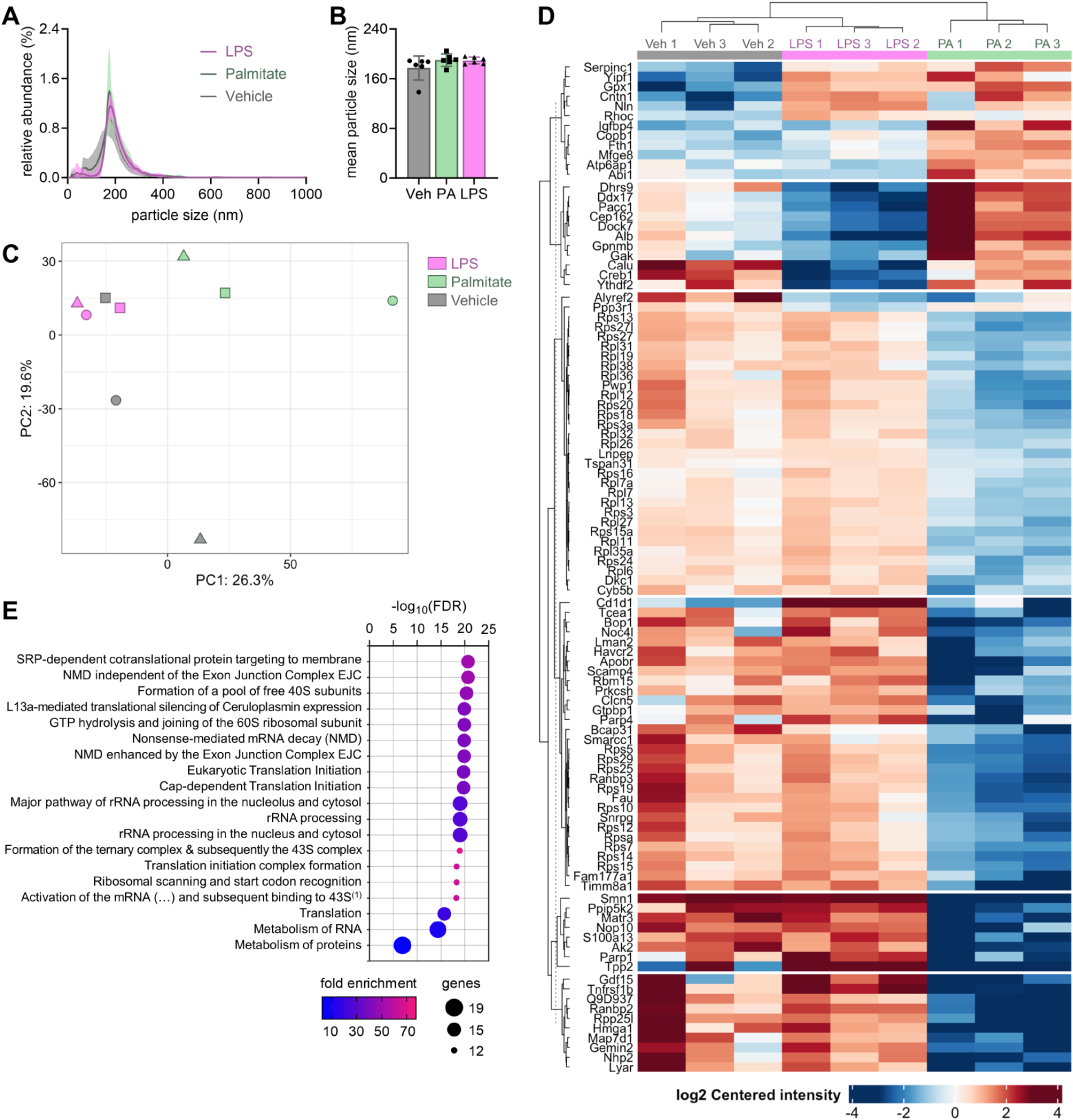
Palmitate alters the proteome of BV2-retreased EVs, including a reduction of proteins involved in RNA processing and protein synthesis. (**A**) Histograms of EV size distribution evaluated by Nanoparticle tracking analysis (NTA) of 6 independent EV isolations, and (**B**) estimated mean particle size for vehicle (Veh), palmitate (PA) and LPS. Data shown as mean ± SD of *n* = 6. (**C**) Score plots of a PCA of the 1000 most abundant proteins in EVs (*n* = 3/group). Each symbol shape represents an independent experiment. (**D**) Heatmap of significant EV proteome differences between either of the 3 experimental groups (fold-change comparisons in supplementary figure S1), and (**E**) significant findings at FDR < 0.01 from the gene ontology analysis of differentially expressed proteins between EVs from palmitate- and vehicle-treated BV2 cells. ^(1)^Full pathway name: “Activation of the mRNA upon binding of the cap-binding complex and eIFs and subsequent binding to 43S”

Palmitate-treated cells did not exacerbate cytokine production, we thus tested whether palmitate could modulate EVs as means of communication to other cells. Cells treated with either palmitate or LPS produced EVs of size like vehicle-treated cells, as determined by NTA (Fig. 3A-B). The estimated amount of EVs released into the 20 mL of media ranged between 3.3 × 10^10^ – 8.9 × 10^11^ for vehicle, 5.2 × 10^9^ – 6.3 × 10^11^ for palmitate, and 1.0 × 10^11^ – 1.1 × 10^12^ for LPS. This suggests that despite higher proliferation, there is no substantial increase in EV release during palmitate exposure.

We then investigated the proteome of microglia-derived EVs in response to each treatment. The abundance of EV markers was larger than that of typical proteins from intracellular compartments (Supplementary Figure S1), in line with the high enrichment of EVs in the preparation. A false discovery rate (FDR) of < 0.01 was set as a threshold protein identification, resulting in a total of 3739 proteins present across a total of 9 experiments (3 per treatment group). From these, only 1636 proteins were present in all analyzed samples. For subsequent analysis, only proteins that were present in at least 2 out of the 3 experiments of each group were kept.

Data was not found missing at random. In fact, EVs from palmitate-exposed cells seemed to lack of proteins that are present in the other groups. Moreover, missing proteins had lower average abundance in samples that did have a measurable signal, suggesting that these are most likely proteins that have very low abundance or are completely absent (true zeroes).

In a PCA using the 1000 most abundant proteins, PC1 allowed to separate palmitate from both LPS and vehicle treatments (Fig. 3C), suggesting that 24 h of exposure to palmitate triggers a unique proteomic signature of released EVs. A differential enrichment analysis revealed significant differences (adjusted *P* < 0.05) in the abundance of 102 proteins in-between either of the 3 comparisons performed (Fig. 3D; supplementary figure S2; data can be explored in Supplementary Table S2). Notably, palmitate-induced alterations on the EV proteome included a reduction in the abundance of several ribosomal proteins and proteins involved in protein metabolism. Indeed, a gene ontology analysis to the EV proteins that were differentially enriched between palmitate and vehicle treatments revealed pathways related to RNA processing, ribosome assembly, and translation processes (Fig. 3E).

### EVs from palmitate-treated cells impact brain function

**Fig. 4:**
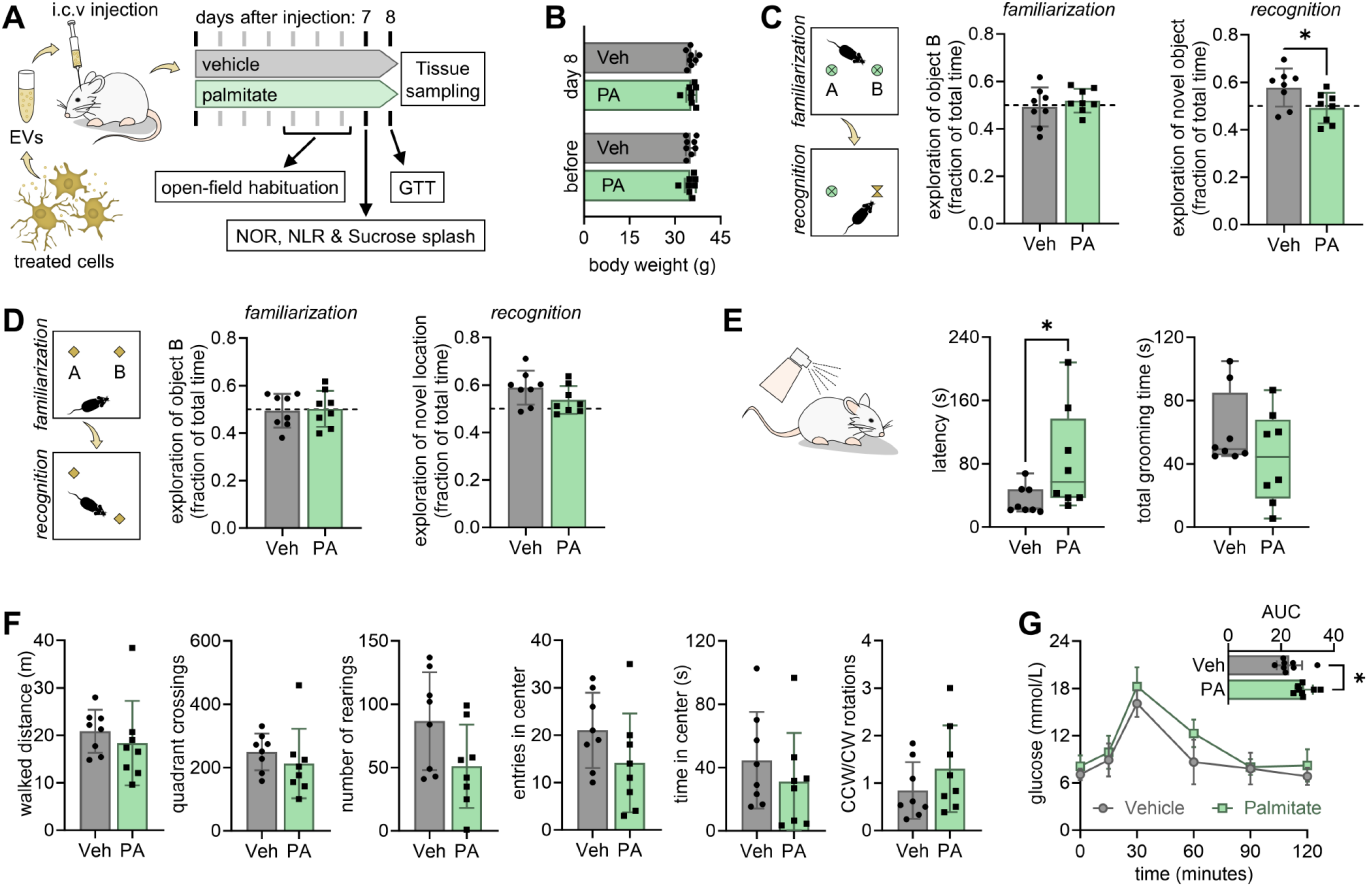
Intracerebroventricular (i.c.v) injection of microglia-derived EVs following palmitate exposure affects cognition and depressive-like behavior, and alters glucose metabolism in mice. (**A**) Mice were injected in the lateral ventricle with EVs (500 ng of protein) collected from BV2 cells after exposure to either palmitate (PA, 200 µmol/L) or vehicle (Veh). (**B**) Body weight of mice before and 8 days after surgery for EV administration. Impaired memory was observed novel object recognition (NOR) test (**C**), and a similar trend was observed in the novel location recognition (NLR) test (**D**). (**E**) Depression-related behavior was assessed by grooming behavior in the sucrose splash test. (**F**) locomotor activity and exploratory behavior assessed in the last habituation day to the open-field arena. (**G**) Glucose clearance in the GTT in the 8th day following EV injection. The inset is the area under the curve (AUC) of the glucose excursion for each mouse. Symbols representing each mouse (*n* = 8) are overlaid on bar graphs showing mean ± SD or box plots showing interquartile ranges. *P < 0.05 from either Student’s t-test or with Mann Whitney test

EVs were obtained from BV2 cells exposed to either palmitate or vehicle, and injected into the lateral ventricle mice, which allows spreading the infusate through the whole brain (confirmed in pilot experiments with trypan blue). A week after injection, we assessed exploratory behavior, memory performance, and depressive-like behavior (Fig. 4A). The i.c.v. injection of EVs had no impact on body weight (Fig. 4B), suggesting good recovery from surgery. In NOR and NOL, neither treatment group showed signs of object bias in the familiarization session (Fig. 4C-D). In the recognition session, mice injected with EVs from vehicle-treated BV2 cells showed preserved memory performance as assessed in the NOR (+ 15%, *P* = 0.029 *versus* random exploration) and NLR (+ 18%, *P* = 0.010 *versus* random exploration). In turn, mice receiving EVs from BV2 cells exposed to palmitate did not show increased exploration of either the novel object (-2%, *P* = 0.710 *versus* random exploration; Fig. 4C) or novel location (+ 7%, *P* = 0.117 *versus* random exploration; Fig. 4C). When comparing memory performance between the treatment groups, significantly lower memory performance between palmitate and vehicle was observed in the NOR task (Fig. 4C).

The sucrose splash test is used to infer on self-care behavior. Mice injected i.c.v. with EVs from palmitate-exposed microglia showed significantly larger latency to start grooming, when compared to vehicle (*P* = 0.037; Fig. 4E). Such reduced self-care behavior is characteristic of depression, although there was no significant change on the total duration of grooming between treatments.

Several parameters of locomotion and exploratory behavior were measured in the empty open field arena, showing no significant differences between the treatments (Fig. 4F). Notably, the ratio of counterclockwise-to-clockwise rotations was also similar between groups and did not show signs of lateralized motor impairment (Fig. 4F).

#### EVs impact glucose homeostasis

EVs injected i.c.v. may impact the hypothalamus, or even reach the blood stream and act on peripheral organs, [22] thus affecting metabolism and glucose homeostasis. Therefore, we performed a GTT after behavior assessments. While baseline glucose was similar between treatment groups (*P* = 0.083), mice receiving EVs from palmitate-exposed BV2 cells showed a larger glucose excursion than the vehicle group, as typified by increased GTT area-under the curve (+ 24%, *P* = 0.018; Fig. 4G).

### EVs can impact microglia in the mouse brain

**Fig. 5:**
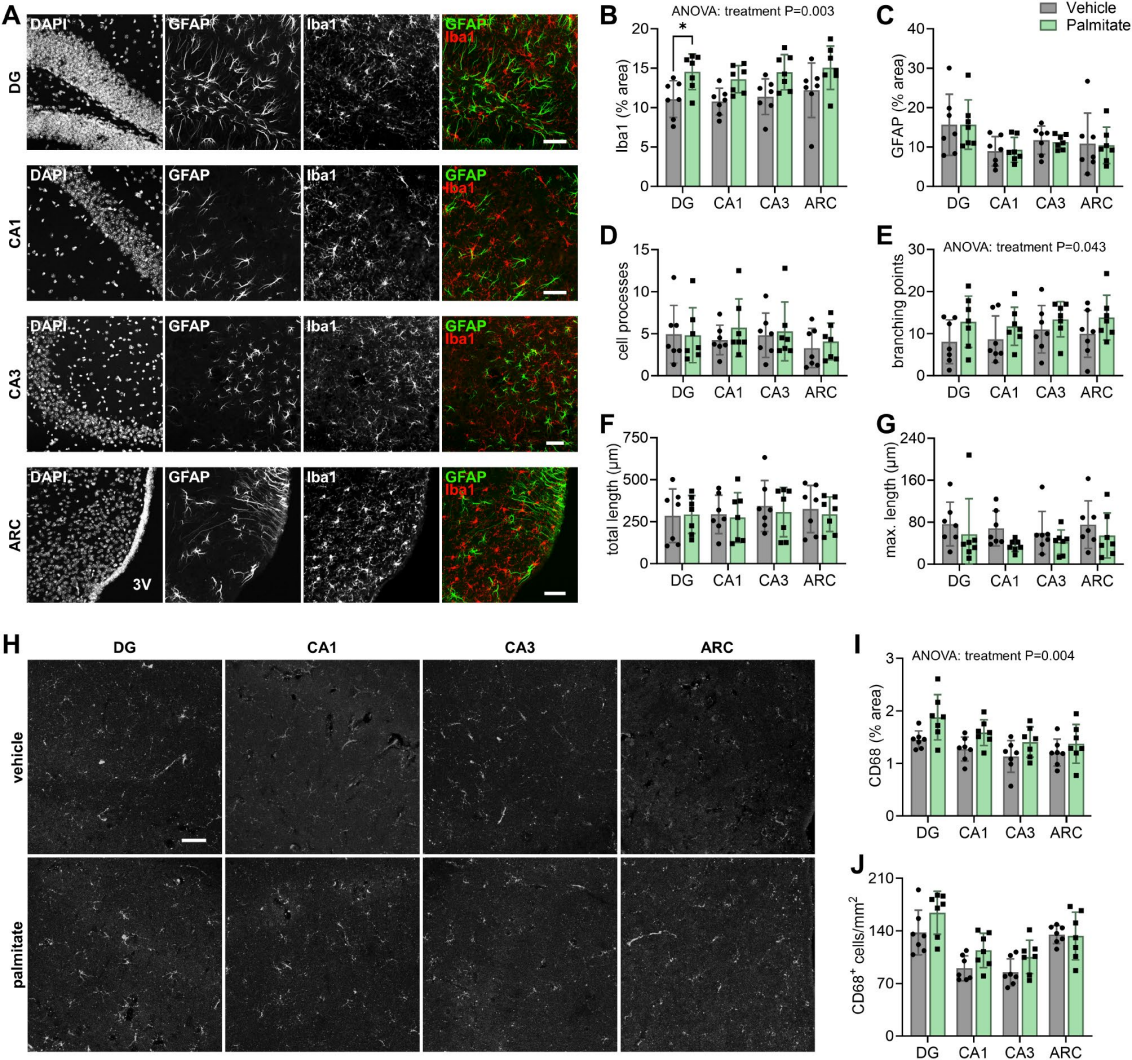
EVs from palmitate-challenged microglia in vitro activate microglia in the hippocampus and hypothalamus of mice in vivo. (**A**) Representative micrographs showing DAPI signal from nuclei, GFAP immunoreactivity in red, and Iba1 immunoreactivity in red in the *dentate gyrus* (DG) and *cornu ammonis* (CA1/CA3) areas of the hippocampus, and *arcuate nucleus* (ARC) of the hypothalamus at 8 days after intraventricular EVs injection. (**B-C**) Fraction of area occupied by iba1 and GFAP immunoreactivity in DG, CA1, CA3 and ARC. (**D**) Mean number of processes, (**E**) number of branching points, (**F**) total cell process length, and (**G**) maximum process length of microglia, as determined from skeleton analysis of 3–4 iba1^+^ cells per mouse. (**H**) Representative micrographs showing CD68 staining at 8 days after EV injection. (**I**) Fraction of are occupied by CD68 immunoreactivity, and (**J**) number of CD68-positive cells. Data is shown as mean ± SD of 7 independent experiments, represented by the individual symbols. *P < 0.05 depicts differences in comparisons following significant effects in ANOVA. Scale bars over the micrographs indicate 50 μm; 3 V = third ventricle

EVs are likely to afford exchange of cellular material in-between microglia, and from microglia to other cells. We then tested whether the injected EVs induced alterations in the morphology of microglia and astrocytes in the mouse hypothalamus (*arcuate nucleus*, ARC), and in the hippocampal *cornu Ammonis* (CA1 and CA3) and *dentate gyrus* (DG) (Fig. 5A). We determined the area covered by immunoreactivity against Iba1 and GFAP, which are microglia and astrocyte markers, respectively. Compared to vehicle, mice treated with EVs from palmitate-exposed BV2 cells showed significantly larger Iba1 coverage across all regions analyzed (ANOVA: region x treatment *F*(3,36) = 0.064, *P* = 0.979; region *F*(3,36) = 1.081, *P* = 0.369; treatment *F*(1,12) = 14.250, *P* = 0.003; Fig. 5B). In contrast, GFAP area was similar in the 2 groups, suggesting that despite microglia expansion, the EVs from palmitate-treated BV2 microglia did not cause overt astrogliosis in vivo (Fig. 5C). Next, we set to explore the morphology of Iba1^+^ cells, that is microglia (Supplementary Figure S3). Microglia from the brain of mice in palmitate and vehicle groups showed similar number of processes sprouting from the cell soma (Fig. 5D). In turn, compared to vehicle, mice injected i.c.v. with EVs from palmitate-exposed cells showed more branching points in the microglia processes (ANOVA: region x treatment *F*(3,36) = 0.142, *P* = 0.934; region *F*(3,36) = 0.546, *P* = 0.654; treatment *F*(1,12) = 5.141, *P* = 0.043; Fig. 5E). Total length and maximum length of the cell processes were similar between groups (Fig. 5F-G). CD68 immunoreactivity was also increased in the brain of mice receiving EVs from palmitate-treated cells, relative to vehicle (ANOVA: region x treatment *F*(3,36) = 0.476, *P* = 0.701; region *F*(2.41,28.96) = 5.397, *P* = 0.007; treatment *F*(1,12) = 12.910, *P* = 0.004; Fig. 5H-I), suggesting larger microglia activation. However, the number of CD68-positive cells was similar between the groups (Fig. 5J).

Our findings indicate that EVs shed by palmitate-exposed microglia induce microglia activation and branching in vivo, despite no overt gliosis after one week of direct administration to the brain.

## Discussion

Our results suggest that microglia respond to palmitate exposure with increased proliferation, and with a metabolic network rearrangement that favors energy production from glycolysis rather than oxidative metabolism, despite increased mitochondria density. In addition, while palmitate did not induce increased cytokine expression, it modified the protein cargo of released EVs which, alone, can contribute to the development of memory impairment, depression-like behavior and glucose intolerance.

### Rearrangement of energy metabolism after palmitate exposure

Microglia are metabolically flexible cells that can utilize a variety of substrates, and their activation upon injury or infection is an energetically costly process [35, 36] When activated by inflammatory stimuli, microglial cells exacerbate glucose uptake and glycolysis [33, 34]. A similar metabolic shift occurs in activated macrophages [37]. Indeed, we have observed this switch from oxidative metabolism to anaerobic glycolysis in BV2 microglia activated by either palmitate or LPS. In contrast, Chausse et al. [38] have reported that BV2 activation by palmitate is sustained by oxidative metabolism, while LPS activation is mostly dependent on glycolysis. It should be noted, however, that Chausse et al. have cultured their cells in the presence of 25 mmol/L of glucose, which is believed to represent hyperglycemic conditions (*e.g*., see Duarte et al. [39] for brain glucose levels observed in the brain of humans and rodents). Primary microglia and BV2 microglial cells express several glucose carriers, with GLUT1 being at least one order of magnitude more abundant than the others, and the one that is upregulated upon microglia activation [40] Our results also support GLUT1 upregulation in both LPS and palmitate exposure.

Activation of BV2 cells and primary microglia by LPS is known to induce mitochondrial fragmentation [41, 42]. Enhanced expression of genes coding for proteins involved in mitochondria fragmentation was not observed in our short (3 h) LPS exposure or upon palmitate treatment. Namely, mRNA levels of mitochondrial fission markers dynamin-related protein 1(Drp1), fission protein 1 (Fis1), and mitochondrial fission factor (Mff) were similar across experimental groups. Instead, we observed a palmitate-induced expression of PGC-1α, which is a key regulator of mitochondrial biogenesis. Indeed, palmitate also induced an increase in mitochondria content, and in the density of proteins belonging to the mitochondrial complexes of the electron transport chain. Together, our findings suggest that although palmitate potentially increases mitochondria biogenesis, it reduces the efficiency of respiration and exacerbates glycolysis. Using ^13^C tracing experiments, we found that palmitate further increases the rate of pyruvate carboxylation, which is needed for *de novo* oxaloacetate synthesis [43], and supports the drainage of Krebs cycle intermediates used in biosynthetic pathways during increased cellular proliferation.

### Lack of palmitate-induced cytokine overexpression

Obesity and saturated fat exposure is known to stimulate NF-κB transcriptional activity and the secretion of pro-inflammatory mediators in peripheral organs, such as liver, white adipose tissue, and leukocytes [44], In the brain, the neuroinflammatory process in DIO remains unclear, and somehow controversial. That might be because microglia reactivity is modulated by specific neurochemical signals within the cell’s microenvironment, and due to transient release of inflammatory mediators. Indeed, there have been reports of both differential microgliosis profiles across distinct brain areas, [45, 46] and biphasic gliosis with transient cytokine release in chronic noxious stimuli, such as that in DIO [7, 11].

We have conducted this study under the assumption that palmitate is the saturated fatty acid in the context of obesity driving neuroinflammation [23]. In fact, palmitate induces much stronger expression of inflammatory factors in primary astrocytes than saturated fatty acids such as laurate or stearate [47]. In addition, 200 µmol/L of palmitate but not stearate impairs mitochondrial function in the C6 astrocyte cell line. [48] In neuronal cell models, both palmitate and stearate can cause cell death. [49] In our hands, while the exposure of BV2 cells to LPS resulted in an enormous expression of pro-inflammatory cytokines, palmitate was devoid of such effect. Accordingly, Chausse et al. [38] also failed to find increased inflammatory mediators in palmitate- or oleate-exposed BV2 cells. Others have suggested that palmitate can even activate anti-inflammatory pathways and reduce TNF-α expression in BV2 microglia [50, 51].

### EVs as neuroinflammatory mediators

DIO leads to depression-like behavior and hippocampal-dependent memory impairment in rodents, although not all diet intervention studies have found pronounced increases in neuroinflammatory markers [6, 11, 14–16, 27, 52, 53]. Microglial inhibition protocols have supported the notion that microgliosis is an important contributor to neuronal dysfunction in the *arcuate nucleus* of the hypothalamus upon DIO, [8] and can both prevent peripheral inflammation and reduce the extent of metabolic syndrome development [54].

However, the exacerbated cytokine production appears to be transient in both the hippocampus and hypothalamus during DIO [7, 11] In later stages of obesity development, in the absence of cytokine overproduction, EVs could contribute with both inflammatory and non-inflammatory molecular messages from microglia to other cells. EVs isolated from palmitate- or LPS-treated cells had unaltered size but displayed significant proteome changes. Surprisingly, our pattern recognition analysis demonstrated larger proteome differences between palmitate and vehicle than between LPS and vehicle groups.

Most significant differentially expressed proteins in palmitate were related to mRNA processing and translation into protein, including the downregulation of several ribosome subunits (Rps5, Rps7, Rps13, Rps14, Rps15a, Rps29, Rpl11, Rpl35a). Ribosome subunits in neurons exhibit a dynamic assembly that is locally regulated in dendrites and synapses [55, 56] Moreover, RNA stress granules and smaller dendritic trees were previously observed when ribosomal proteins were depleted from neurons with already established dendrites [57]. Thus, it is likely that microglia EVs deliver components of the ribosomal machinery to support protein synthesis in neuronal processes, and we speculate that the reduction in such components within microglial EVs after palmitate exposure can result in alterations of synaptic plasticity and neuronal connectivity. Decreased expression of ribosomal proteins has been reported in metabolically healthy obese individuals, [58] including Rps29 and Rpl35a, also found to be downregulated in the present study. Tardif et al. further reported that palmitate exposure induced endoplasmic reticulum stress and suppress protein synthesis while upregulating proteolytic systems in skeletal muscle [59]. Since modulation of ribosomal activity and translation is a key determinant of innate immune gene translation in microglia, [60] we speculate that reduced levels of ribosome-associated components in EVs after palmitate exposure might also contribute to refrain protein translation in target microglia. Such mechanism of control of innate immune response in resident microglia would explain why limited microgliosis is found in diet-induced obesity models [12–16].

Previous reports demonstrated that EVs from microglia activated by ATP, IFN-γ or LPS can impact neurons and astrocytes [61, 62]. Namely, exosomes from IFN-γ/LPS-activated microglia trigger dopaminergic neurodegeneration, [61] and EVs secreted from ATP-stimulated microglia induce stronger astrocyte activation with upregulation of IL-10 and IL-6 expression, compared to constitutive EVs. [62] In our experimental setup, when EVs were given i.c.v., mice receiving EVs from palmitate-treated microglia developed memory impairment, as assessed by the lower capacity to recognize the novelty in the NOR test. An increase in latency to grooming in the sucrose splash test further indicated a depression-like behavior. Moreover, mice injected with EVs derived from palmitate-exposed microglia developed glucose intolerance, when compared to the vehicle group, suggesting alterations in central regulation of metabolism.

In DIO, impaired glucose homeostasis occurs before insulin resistance is installed, [15, 63] which is in line with direct actions of EVs on hypothalamic glucose-sensing neurons. However, one must keep in mind that smaller brain-born EVs, such as exosomes, reach the systemic circulation, [64] and could act on peripheral organs as well.

Finally, we investigated whether EVs alone could be mediators of neuroinflammation. Thus, we evaluated gliosis in the mouse brain following EV administration, with focus on the hippocampus that controls learning and memory, and the hypothalamus that is the main central glucose sensing area. Notably, we have observed some degree of microgliosis but not astrogliosis when injecting i.c.v. the EVs from palmitate-exposed microglia, relative to EVs from vehicle experiments. The absence of astrogliosis is somehow surprising since microglia-derived EVs after ATP stimulation were found to modulate astrocytes [62].

### Limitations

We have characterized energy metabolism of BV2 cells but have not performed in-depth investigations into the mechanisms that drive mitochondrial alterations, which could include intracellular accumulation of lipids, and the impairment in insulin signaling. Moreover, although this study puts forward novel ideas for further testing, it is important to note that we have studied the BV2 microglial cell line and not primary microglia, although we know they tend to behave similarly [65, 66]. The cell line allowed us to have access to larger amounts of material (namely EVs) without collecting tissue from large numbers of animals, but our findings might not fully represent microglia responses in vivo. In addition, we have studied protein content of EVs, but not other types of cargo, such as small metabolites, lipids, and nucleic acids. In fact, it is thought that EVs carry microRNAs that control the expression of a variety of proteins, namely proteins involved in synaptic pruning, energy metabolism, immune response, and translation/transcription [18, 67, 68]. Whether these microRNAs are present and modified after palmitate exposure was not tested, but further studies on this topic are warranted.

The concentration of palmitate used in our study is in the range of that used in many other studies on cell cultures, which is at least 10-fold larger than that found in the cerebrospinal fluid (CSF) of humans (5–20 µmol/L). [70, 71]. However, stearate a more abundant saturated fatty acid in the human CSF, ranging from 17 to 120 µmol/L [70, 71] .Together, these two main brain free fatty acids appear at concentrations in the same order of magnitude of our study. Melo et al. reported that obese individuals show a 2-fold increase in CSF palmitate but have not reported changes in levels of stearate [23].

We have tested the injection of EVs in young mice, although metabolic disease is expected to be a risk factor mainly in elderly individuals. [1–3] Published research shows that a very short exposure to a high-fat diet is already sufficient to impact the brain of mice at 2–3 months of age. Thus, we decided to test whether microglia-derived EVs exposed to palmitate mimic such effects at this age. Notably, microglia responses have been shown to vary with age, including metabolism, cytokine release, the important phagocytic capability and the expression of various pattern recognition receptors to regulate migration [72–74]. Therefore, one should indeed expect differences in activation and responses of resident microglia at young and old age.

## Conclusion

In this study, using BV2 microglia, we demonstrate that palmitate exposure does not drive expression of cytokines that is typical of microglia activation. Instead, palmitate led to the release of EVs with modified protein cargo that, alone, is sufficient to induce some degree of microgliosis in the mouse, and to impact brain function. The EV-mediated palmitate dysfunction is thus a plausible mechanism by which microglia participate in brain dysfunction during DIO.

### Electronic supplementary material

Below is the link to the electronic supplementary material.

Supplementary Material 1

Supplementary Material 2

Supplementary Material 3

Supplementary Material 4

Supplementary Material 5

## Data Availability

Data is provided within the manuscript or supplementary information files.
