# Supplementary figures and images for: Extracellular vesicles released from microglia after palmitate exposure impact brain function
Source: J Neuroinflammation. 2024 Jul 16;21:173. doi: 10.1186/s12974-024-03168-7 (PMC11253458; doi:10.1186/s12974-024-03168-7)

## Slide 1
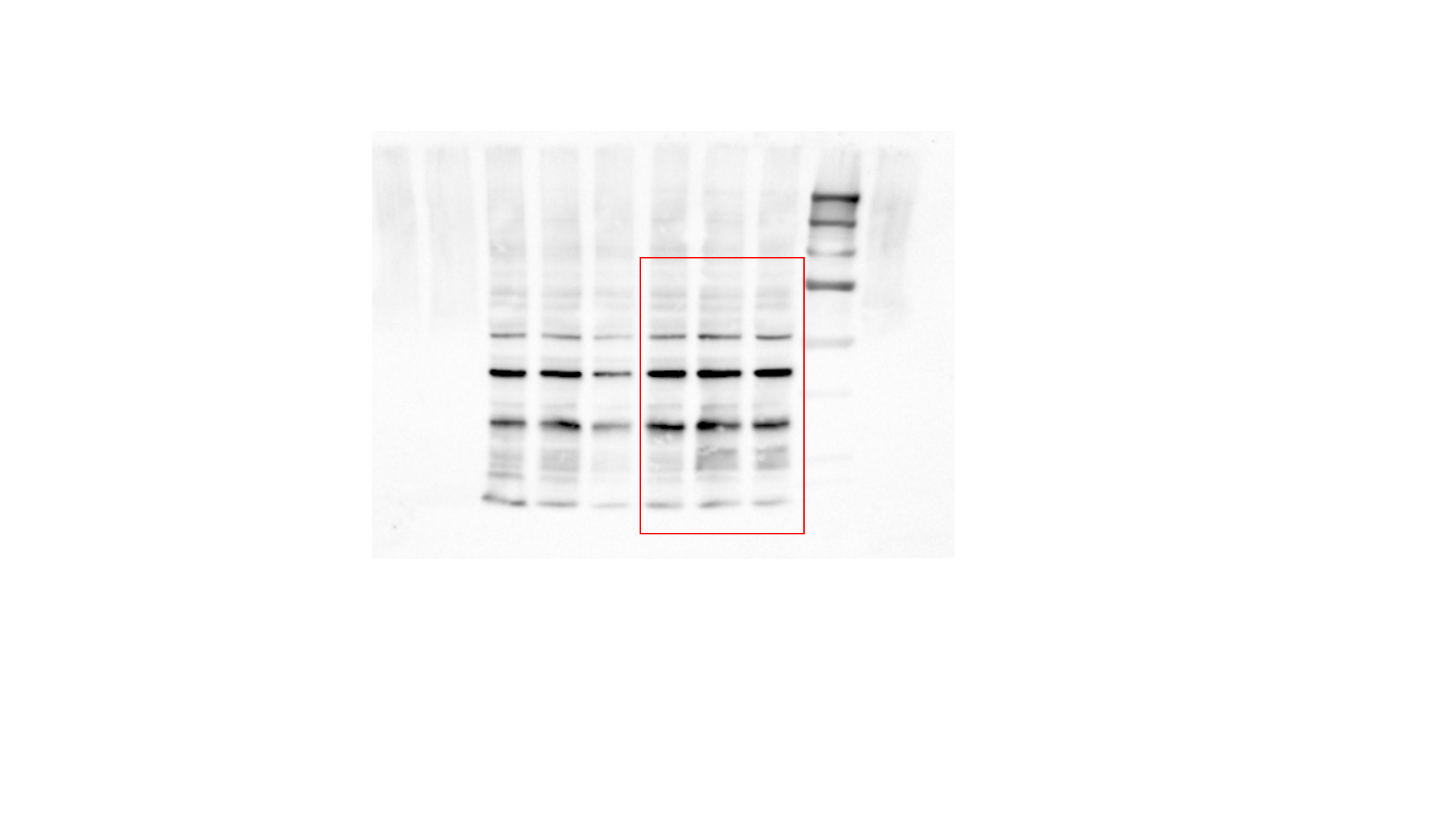

Supplement: Supplementary file 2 — Supplementary Material 2 [file 12974_2024_3168_MOESM2_ESM.pptx]
