# Supplementary material for: Extracellular vesicles released from microglia after palmitate exposure impact brain function
Source: J Neuroinflammation. 2024 Jul 16;21:173. doi: 10.1186/s12974-024-03168-7 (PMC11253458; doi:10.1186/s12974-024-03168-7)
Supplement: Supplementary file 5 — Supplementary Material 5 [file 12974_2024_3168_MOESM5_ESM.pdf]

Supplementary material for de Paula *et al.*

Supplementary figures

Figure S1. Relative abundance of EV markers compared to 4 proteins abundant in mitochondria, Golgi apparatus and endoplasmic reticulum, or nucleus. Tomm20 and Golga5 were not detected in 6 and 3 samples respectively. Other markers can be explored in Table S3.

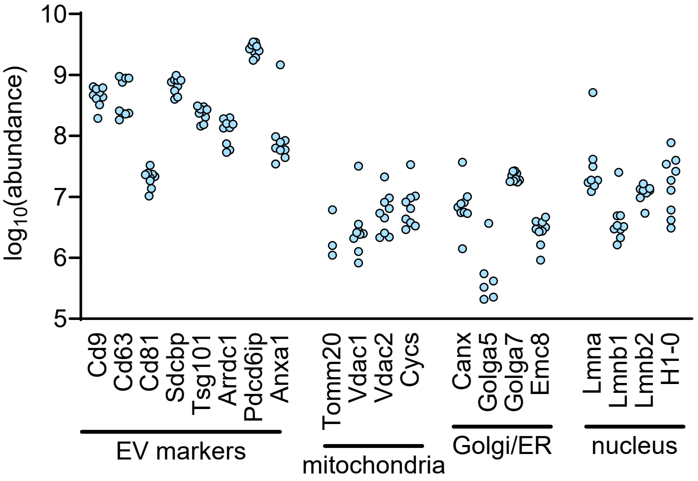

Figure S2. Volcano plots for EV proteome differences between cells treated with LPS (lps), palmitate (pa) or vehicle (veh).

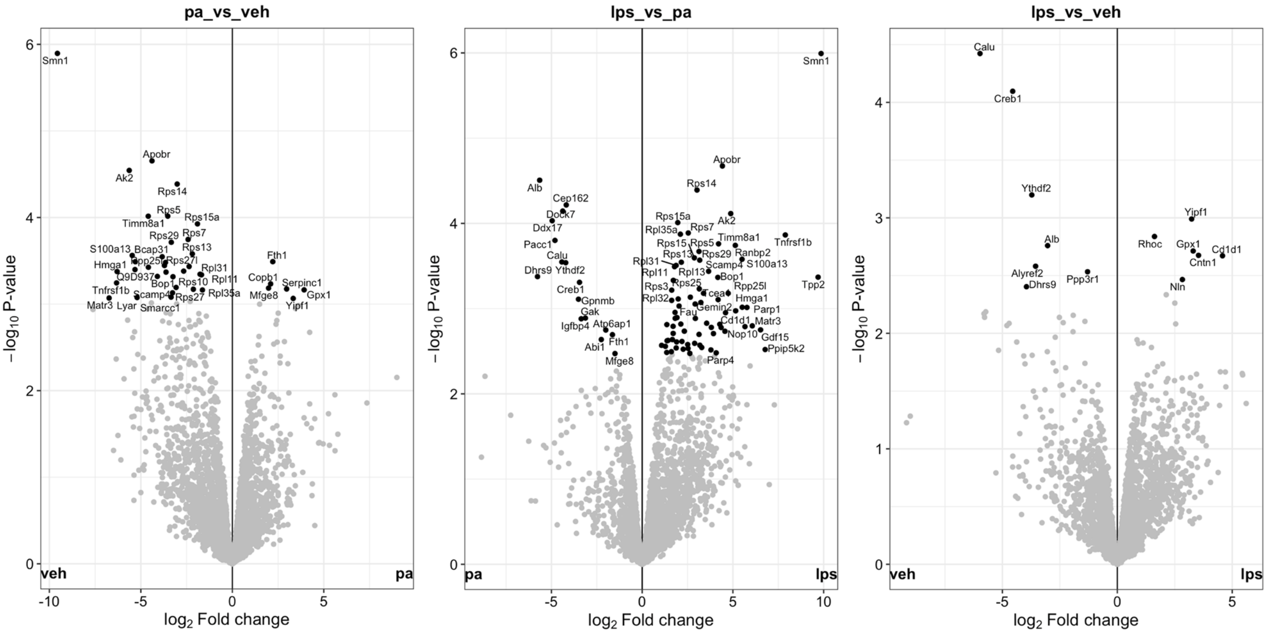

Figure S3. Microglia branching analysed 7 days after i.c.v. injection of EVs from microglia treated with palmitate or vehicle. Representative analysis of two microglia in the mouse *dentate gyrus*, CA1 and CA3 of the hippocampus, and in the *arcuate nucleus* of the hypothalamus.

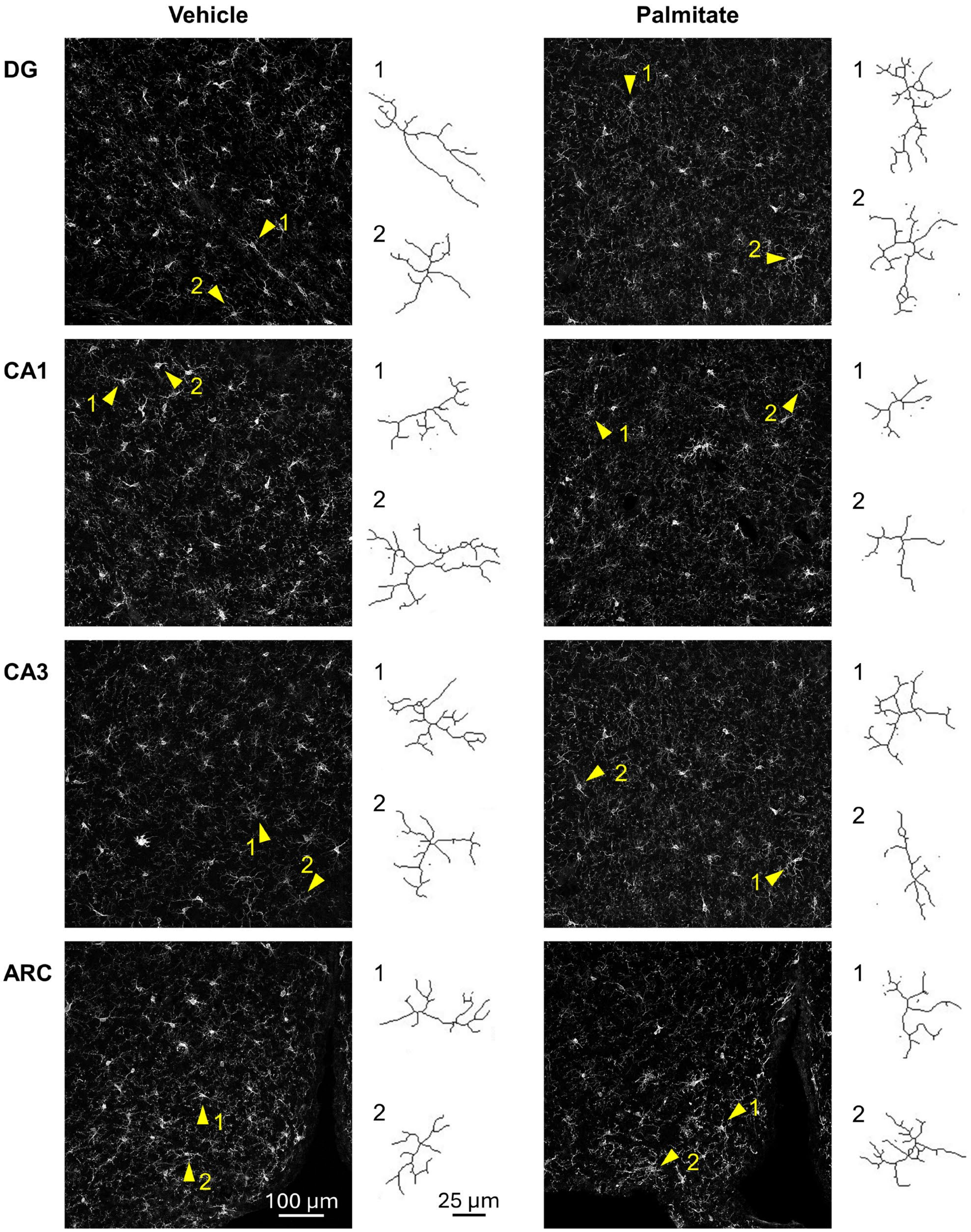

Supplementary files

Table S1. Results from ANOVA statistics

Table S2. Differential expression testing results following comparison of proteomes of EVs released from microglia exposed to vehicle, palmitate and LPS.

Table S3. Proteomic data from 3 samples of EVs released from microglia exposed to vehicle, palmitate and LPS.

Supplementary methods

Palmitate preparation

A stock solution of sodium palmitate was prepared by conjugation with bovine serum albumin (BSA). Briefly, 4.54 g of fatty acid-free BSA (#A7030, Sigma-Aldrich, St. Louis, MO-USA) was dissolved at 36ºC in 16 mL of 150 mmol/L NaCl, and 61.2 mg of sodium palmitate (Sigma-Aldrich #P9767) was dissolved at 71ºC in 4 mL of 150 mmol/L NaCl. The palmitate solution was then slowly added to the BSA solution while stirring, filtered with a sterile 22-µm polyvinylidene fluoride filter (Sigma-Aldrich #LGVV255F), and frozen at -20 ºC in 11 mmol/L palmitate aliquots.

OCR assay

Cells were washed and incubated for 1 hour with 180 µL assay medium [in mmol/L: 5 glucose (Agilent #103577-100), 1 pyruvate (Agilent #103578-100), 2 glutamine (Agilent #103579-100) in XF DMEM medium (Agilent #103575-100)] in atmospheric air at 37°C. After equipment calibration, baseline respiration measurements were followed by 1.5 µmol/L oligomycin addition to determine ATP-linked and proton leak-driven respiration. The mitochondrial uncoupler FCCP (carbonyl cyanide-p-trifluoromethoxy-phenylhydrazone, 0.5 µmol/L) was added to induce maximal respiratory capacity. Non-mitochondrial respiration was determined after the addition of 0.5 µmol/L rotenone plus 0.5 µmol/L antimycin A (inhibitors of complex I and complex III, respectively).

ECAR assay

Cells were incubated in assay medium without glucose or pyruvate (sodium bicarbonate and

FBS were absent) in atmospheric air at 37 °C. Baseline ECAR was measured after addition of 10 mmol/L glucose. The conversion of glucose to pyruvate, and production of lactate that is released with protons leads to medium acidification that can be used as surrogate of glycolysis. Oligomycin (1 µmol/L) was added to inhibit mitochondrial ATP production, revealing the cellular maximum glycolytic capacity. Non-glycolytic ECAR was measured after addition of the glycolysis inhibitor 2-deoxy-D-glucose (2-DG, 50 mmol/L).

Nuclear magnetic resonance (NMR) spectroscopy

Dried samples were re-suspended in 100 mmol/L sodium phosphate buffer pH 7.4 prepared in $^{2}H_2O$ (>99.9%, Sigma-Aldrich), containing 0.01% NaN_3. Sodium fumarate (0.3 µmol) was added as internal standard, and samples were transferred into 5 mm Wilmad NMR tubes (Sigma-Aldrich).

NMR spectra were acquired on a Avance III HD 600 MHz spectrometer with a standard TCI cryoprobe (Bruker Nordic, Solna, Sweden). Solvent-suppressed ^{1}H-NMR spectra were acquired with the ZGPR pre-saturation pulse sequence with spectral width of 9 kHz, 3 s acquisition time, a relaxation delay of 22 s, and 24 scans per cell extract. ^{1}H-decoupled ^{13}C-NMR spectra were acquired using the ZGPG30 sequence with 30 kHz spectral width, 2 s acquisition time, and a relaxation delay of 2 s. To achieve adequate signal-to-noise ratio, ^{13}C spectra were recorded with at least 30,000 scans.

Mass spectrometry (MS) for proteomics

EVs (20 µL) were mixed with 30 µL of RIPA buffer (Sigma-Aldrich #R0278), and sonicated with 30 cycles of 15 s ON-OFF using a BioRuptor (Diagenode, Denville, NJ-USA). The EV lysate was reduced with 10 mmol/L dithiothreitol at 56 °C for 30 minutes, followed by alkylation with 20 mmol/L iodoacetic acid for 30 minutes in the dark. Samples were precipitated with ice-cold ethanol 90%(v/v) overnight at -20 °C. Samples were centrifuged at 14,000 x g for 10 minutes. The pellets were air-dried, re-dissolved in 50 µL 100 mmol/L ammonium bicarbonate, sonicated, and centrifuged again. Supernatants were collected, and protein concentration was determined using a DeNovix nanospectrophotometer (AH diagnostics, Solna, Sweden). Protein samples (15 µg) were digested overnight at 37 °C with trypsin (Promega, Madison, WI-USA) in a protein:trypsin ratio of 50:1 (w/w). The digestion was stopped by 5 µL 10%(v/v) trifluoroacetic acid. Samples were dried using a SpeedVac and re-dissolved in a mixture of 2%(v/v) acetonitrile and 0.1%(v/v) trifluoroacetic acid.

Samples were analyzed in an Orbitrap Eclipse Tribrid mass spectrometer coupled with an Ultimate 3000 RSLCnano system (ThermoFischer). The HPLC used a two-column setup: peptides were loaded into an Acclaim PepMap 100 C18 pre-column (75 µm x 2 cm; ThermoFischer) and then separated with the flow rate 300 nL/min in an EASYspray column (75 µm x 25 cm, nanoViper, C18, 2 µm, 100 Å; ThermoFischer). The column temperature was set 45 °C. Peptides were eluted with a nonlinear gradient using 0.1%(v/v) formic acid in water as solvent A, and 0.1%(v/v) formic acid in 80%(v/v) acetonitrile as solvent B. Solvent B was maintained at 2% during 4 minutes, increased to 25% during 100 minutes, to 40% during 20 minutes, to 95% during 1 minute, and finally kept at 95% for 5 min to wash the column.

Samples were analyzed with the positive data-dependent acquisition (DDA) mode. The full MS resolution was set to 120,000 at normal mass range, and the automatic gain control target (AGC) was set to standard with the maximum injection time to auto. The full mass range was set 350-1400 m/z. Precursors were isolated with the isolation window of 1.6 m/z and fragmented by HCD with the normalized collision energy of 30. MS^2 was detected in the Orbitrap with the resolution of 15,000, and AGC and maximum injection time were set to standard and 50 ms, respectively.

The raw DDA data were analyzed with Proteome Discoverer 2.5 Software (ThermoScientific), and the peptides were identified using SEQUEST HT against the UniProtKB Mouse database (UP000000589) with the following parameters applied: cysteine carbamidomethylation as static modification, and *N*-terminal acetylation and methionine oxidation as dynamic modification. Precursor tolerance was set to 10 ppm, and fragment tolerance was set to 0.05 ppm. Up to 2 missed cleavages were allowed. Percolator false discovery rate (FDR) was used for peptide validation at a q-value below 0.01. The extracted chromatographic intensities were used to compare peptide abundance across samples.

Injection of EVs in the lateral ventricle

Mice were anesthetized with isoflurane (induction with 5%; maintenance with 2-3% in air), and their heads were fixed at a 45° angle in a stereotactic frame (Kopf Instruments, Tujunga, CA-USA) and body placed on a heating pad. After craniotomy under magnification, a glass micropipette (diameter 20-40 µm) was used to deliver 500 ng protein from fresh BV2-derived EVs to the lateral ventricle (anterior/posterior, 0.34 mm from bregma; medial/lateral, 1.0; dorsal/ventral, -2.2 mm from the skull; injection site confirmed in pilot experiments injecting trypan blue). EVs were delivered in multiple microinjections over 3 minutes using air pressure

from a PLI-100A Pico-Injector (Harvard Apparatus, Cambridge, UK), and were allowed to diffuse during 4 minutes before the needle was withdrawn. After, animals received subcutaneous saline for hydration (1 mL), and 5 mg/kg Bupivacaine (Marcain, Aspen Nordic, Ballerup, Danmark) for pain relief upon recovery.
